# Characterization and compilation of polymorphic simple sequence repeat (SSR) markers of peanut from public database

**DOI:** 10.1186/1756-0500-5-362

**Published:** 2012-07-20

**Authors:** Yongli Zhao, Channapatna S Prakash, Guohao He

**Affiliations:** 1 Department of Agricultural and Environmental Sciences, Tuskegee University, Tuskegee, AL, 36088, USA

**Keywords:** SSR, Motif, Polymorphism, Cultivated peanut

## Abstract

**Background:**

There are several reports describing thousands of SSR markers in the peanut (*Arachis hypogaea* L.) genome. There is a need to integrate various research reports of peanut DNA polymorphism into a single platform. Further, because of lack of uniformity in the labeling of these markers across the publications, there is some confusion on the identities of many markers. We describe below an effort to develop a central comprehensive database of polymorphic SSR markers in peanut.

**Findings:**

We compiled 1,343 SSR markers as detecting polymorphism (14.5%) within a total of 9,274 markers. Amongst all polymorphic SSRs examined, we found that AG motif (36.5%) was the most abundant followed by AAG (12.1%), AAT (10.9%), and AT (10.3%).The mean length of SSR repeats in dinucleotide SSRs was significantly longer than that in trinucleotide SSRs. Dinucleotide SSRs showed higher polymorphism frequency for genomic SSRs when compared to trinucleotide SSRs, while for EST-SSRs, the frequency of polymorphic SSRs was higher in trinucleotide SSRs than in dinucleotide SSRs. The correlation of the length of SSR and the frequency of polymorphism revealed that the frequency of polymorphism was decreased as motif repeat number increased.

**Conclusions:**

The assembled polymorphic SSRs would enhance the density of the existing genetic maps of peanut, which could also be a useful source of DNA markers suitable for high-throughput QTL mapping and marker-assisted selection in peanut improvement and thus would be of value to breeders.

## Findings

### Background

Cultivated peanut (*Arachis hypogaea* L.) is among the most important legume crops and a valuable source of oil and protein. Grown on six continents, it is economically the second most important legume in the U.S. Peanuts are planted annually on about 22 million ha worldwide, with a production of 35 million tons (source: http://www.agrostats.com/world-statistic/world-peanut.html).

Peanut is a self-pollinated allotetraploid (2n = 4x = 40) crop with a large genome (2.8 Gbp). Unlike many other polyploid crop species, cultivated peanut is generally believed to be monophyletic in origin [1]. Thus, peanut germplasm exhibits far less molecular genetic variation than most other cultivated crops resulting in the detection of fewer DNA markers in this crop. Consequently, marker-assisted selection, an important tool now in the improvement of many crops, is yet to play a significant role in peanut breeding. Paucity of DNA markers has also resulted in inadequate understanding of the nature and evolution of the peanut genome.

During the past two decades, much effort has been made to develop genetic and genomic tools in cultivated peanut, such as construction of BAC libraries [2,3], cDNA libraries [4-7], genetic linkage maps [8-18], and development of DNA markers [19-36]. Among various molecular markers investigated so far, simple sequence repeats (SSR) have emerged as the preferred DNA marker system for conducting genetic and genomic studies in cultivated peanut [10,11,18,23,26-28,32,33]. To date, nearly 10,000 SSRs have been identified by various research groups around the world. Initial development of SSR markers in peanut employed DNA fragments containing SSRs enriched from genomic libraries by using various SSR probes. Currently SSRs are increasingly developed through data mining of EST and BAC-end sequences. While there are 32 publications on peanut DNA markers so far, there is a need to analyze all existing SSR markers in peanut to develop a central database of polymorphic SSRs with unambiguous labels gleaned from published literature and the public genome database. Such a comprehensive review of polymorphic SSRs would help to advance peanut research and improvement as it would provide an overall snapshot of all existing DNA markers as well as those that are polymorphic. Further, there is considerable interest among peanut breeders to introduce useful genes from wild species to improve genetic diversity using marker-assisted selection using polymorphic markers.

### Methods

Information on publicly available peanut SSRs was collected by scanning scientific publications. Based on sequence similarity search with legacy *Arachis* SSR primer sequences, redundant primer sequences were detected by BLAST with an E-value cut off of 1e^-20^. DNA sequences containing polymorphic SSRs were re-searched for motif and repeat number using SSRIT software. Polymorphic SSRs as well as their polymorphism information content (PIC) values were collected from original and cited publications, or determined by laboratory testing for polymorphism using a panel of cultivated peanut genotypes by the authors. These eight cultivated varieties viz., Tifrunner, GT-C20, SunOleic 97R, NC94022, Yue you 92, Xin Hui Xiao Li, D99, and H22 are also parental genotypes of four mapping populations. Genomic DNAs were extracted from these genotypes using MasterPure Plant Leaf DNA Purification Kit (Epicentre, Madison, WI). The PCR program was subject to 94°C/3 min for initial denaturation, followed by 35 cycles of 94°C/30 sce, 55°C/30 sec, and 72°C/30 sec, and 72°C/5 min for final extension. PCR products were resolved in polyacrylamide gel in LI-COR 4300 DNA Analyzer (LI-COR, Lincoln, NA). All polymorphic SSRs were listed in the Microsoft Excel file as a reference and GenBank accession numbers were included wherever available in order to track their original flanking sequences by hyperlink. SSRs mapped in published genetic linkage maps were highlighted by authors’ name. Resources of species and DNA domains from which SSRs were identified were also shown to indicate genomic and EST-SSRs, or cultivated and wild species SSRs.

### Findings

Redundancy of SSRs developed from different research groups along with the use of non-uniform marker names have resulted in duplicate genotyping of peanut germplasm and inefficient use of resources in peanut genomics. Therefore, there is a need for central depository of informative SSR markers for peanut including all published markers but without redundancy by employing unique and unambiguous marker names. We have attempted to develop such a set of polymorphic SSR markers in peanut.

The total number of SSRs reported to date in both cultivated and wild peanut species from the published literature was 9,274 (Table 1). From these, we identified 1,343 SSR markers (14.5% of the total) that detected variation within peanut germplasm. We further analyzed these polymorphic SSRs to gain insights into their nature and frequency. All published SSRs were summarized in Table 1, which shows the source, name, and numbers of developed, polymorphic and mapped SSRs. The length of most sequences was ranged from 100 to 500 bp. Assuming the average length of SSR containing sequences is 250 bp, these SSRs would contain 2.3 Mbp which corresponds to 0.083% of the peanut genome (2,800 Mbp). Among these SSRs, 5,946 were EST-SSRs and 3,328 were genomic SSRs, from which 603 and 740 were confirmed to be polymorphic at frequencies of 10.1% and 22.2% from EST and genomic sequences, respectively.

**Table 1:** List of total publicly available and polymorphic SSR markers in peanut

**Marker name (prefix)**	**EST or genomic SSR**	**Total no of SSRs developed**	**No of polymorphic SSR**	**No of mapped SSR**	**Publication**
Ah4-xx, Ah6-xx	Genomic	26	6	4	[23]
Apxx	Genomic	7	2	1	[25]
PMxx	Genomic	275	43	33	[26,27]
					[35]
PMxx	EST	44	5	4	[27]
pPGPseqxx, pPGSseqxx	Genomic	226	140	93	[28]
Ah-xx	Genomic	67	12	7	[29]
Ah1xx, Ah2xx, gi-xx	Genomic	121	84	75	[10]
AS1RNxx, AS1R1xx, AS1MLxx, gi-xx	EST	112	20	12	[10]
GAxx	Genomic	103	46	35	[30]
AS1RNxx, AS1RMxx	EST	107	14	7	[5]
Ahxx	Genomic	13	9	7	[37]
S-xx	Genomic	123	45	4	[31]
EM-xx, EE-xx	EST	290	29	9	[6]
IPAHMxx	Genomic	170	54	36	[32]
GMxx	EST	2,138	156	133	[34]
AHMxx	Genomic	2	2	0	[38]
AHBGSxx	EST	35	2	0	[11]
ICGMxx	Genomic	23	8	0	[33]
Fxx, PDxx	EST	33	4	0	[39]
AHSxx	EST	3,187	373	9	[7]
					[18]
GNBxx	Genomic	1,152	167	79	[18]
Adxx, Aixx	Genomic	167	13	10	[18]
AHGSxx	Genomic	706	23	17	[18]
Ah3xx	Genomic	147	86	18	[36]
Total		9,274	1,343(14.5%)	593(6.4%)	

Additional file 1 provides descriptive information on the polymorphic SSR markers. This file contains other informations, such as, marker name, primer name, alternative name, and GenBank accession numbers where they were available. These polymorphic SSRs were identified by various research groups around the world and often employed different names to denote the same SSR marker. In some instances, two different markers have very similar names, adding to the confusion; for example Ah-xx developed by [29] and Ahxx by [37], sound similar but are from different citations.

Some markers having unique names such as marker IPAHMxx and XIPxx, are in fact the same markers but can be easily mistaken as different markers. Further, some marker names and their primer names are often referred to as if they are different markers, such as marker name Ah1TC3A12 with primer name TC3A12, both of which could be mapped on the same genetic linkage map. In the Additional file 1, we present a list of such redundant markers in effort to eliminate duplicate naming of markers. All polymorphic SSR markers listed in the Additional file 1 provide clear information of their source, origin and nature. We believe that such a snap-shot of information on all the available polymorphic SSRs in peanut will serve as a useful resource for high-throughput genotyping by array-based platforms in QTL mapping and marker-assisted selection in peanut breeding.

Among 1,343 polymorphic SSR markers, dinucleotide and trinucleotide motifs were the most predominant and a few were the others. The predominant 1,508 di- and tri-numcleotide motifs were identified and sorted as EST-SSRs or genomic SSRs (Table 2). EST sequences harbored 597 SSR motifs in which motifs AAG (21.1%) and AG (20.9%) were most abundant. Genomic SSRs had 911 motifs where motif AG was the most abundant (comprising 46.7%) followed by motifs AT (13.6%), AC (12.3%), and AAT (12.0%). The detection of such higher percentage of motif AG in genomic sequences might be because of the bias stemming from the use of dinucleotide SSRs as probes, such as (AG)n in the enrichment approach for identification of SSRs in the peanut genome [26]. Interestingly, with one exception no EST-SSR or genomic SSR with motif CG was detected polymorphic. A similar result was reported by Moretzsohn et al. 2005 [10]. In total, motif AG (43.9%) was the most polymorphic and frequent SSR marker type derived from both EST-SSRs and genomic SSRs, followed by AT (12.4%), AAT (11.1%), AC (10.6%), and AAG (9.1%). These are also the motifs that are generally most abundant in the peanut genome [18,28], while in the soybean genome, motifs AT, AAT, and AAAT were the most abundant after searching whole genome sequences [39]. 

**Table 2:** Distribution of various types of motifs in polymorphic EST-SSRs and genomic-SSRs

**Motif**	**EST-SSR (%)**	**Genomic-SSR (%)**	**Total (%)**
AT/TA	31 (5.2)	124 (13.6)	155 (10.3)
AG/GA/CT/TC	125 (20.9)	425 (46.7)	550 (36.5)
AC/CA/TG/GT	18 (3.0)	112 (12.3)	130 (8.6)
GC/CG	1 (0.2)	0 (0.0)	1 (0.1)
AAG/AGA/GAA/CTT/TTC/TCT	126 (21.1)	57 (6.3)	183 (12.1)
AAT/ATA/TAA/ATT/TTA/TAT	55 (9.2)	109 (12.0)	164 (10.9)
ATG/TGA/GAT/CAT/ATC/TCA	47 (7.9)	18 (2.0)	65 (4.3)
AAC/ACA/CAA/GTT/TTG/TGT	46 (7.7)	34 (3.7)	80 (5.3)
ACC/CCA/CAC/GGT/GTG/TGG	32 (5.4)	10 (1.1)	42 (2.8)
AGG/GGA/GAG/CCT/CTC/TCC	35 (5.9)	4 (0.4)	39 (2.6)
AGT/GTA/TAG/ACT/CTA/TAC	9 (1.5)	5 (0.5)	14 (0.9)
AGC/GCA/CAG/GCT/CTG/TGC	30 (5.0)	4 (0.4)	34 (2.3)
ACG/CGA/GAC/CGT/GTC/TCG	14 (2.3)	2 (0.2)	16 (1.1)
GGC/GCG/CGG/GCC/CCG/CGC	28 (4.7)	7 (0.8)	35 (2.3)
Total	597	911	1,508

Comparison of the length of SSRs revealed that the mean length of dinucleotide SSRs was significantly longer than those in trinucleotide SSRs for EST-SSRs and genomic SSRs, respectively (t = 12.48 and t = 8.79, p < 0.0001) (Table 3). This finding was consistent with observation in barley [40], sugarcane [41] and soybean [42]. As the frequency of polymorphism was compared, SSRs derived from genomic sequences was significantly higher than EST-SSRs in dinucleotide SSRs, but was lower than in trinucleotide SSRs using Fisher’s exact test (P < 0.0001). 

**Table 3:** Comparison of motif number and mean of repeat number between dinucleotide and trinucleotide SSRs

**Motif**	**EST-SSR**		**Genomic SSR**	
	**No of motifs**	**Repeat number Mean ± SD**	**No of motifs**	**Repeat number Mean ± SD**
Dinucleotide	175	11.35 ± 5.69	661	16.50 ± 8.88
Trinucleotide	422	6.94 ± 2.79	250	11.05 ± 6.95

Many studies have reported that SSRs with longer repeat length are more polymorphic in plant species [10,18,43,44]. In this study, longer mean length of SSR repeat was found in dinucleotide SSRs, but they exhibited higher polymorphism frequencies as trinucleotide SSRs in EST-SSRs. This may be due to changes of dinucleotide repeat length in exons that are likely to be suppressed due to the deleterious nature of the frame-shift mutation that would frequently result in translated regions [42,45]. Expansion or contraction of SSR repeat length can occur because of replication slippage which is considered as one of the main reasons for SSR mutations. SSR instability is also dependent on motif size, nucleotide content and SSR length [46].

**Figure 1:**
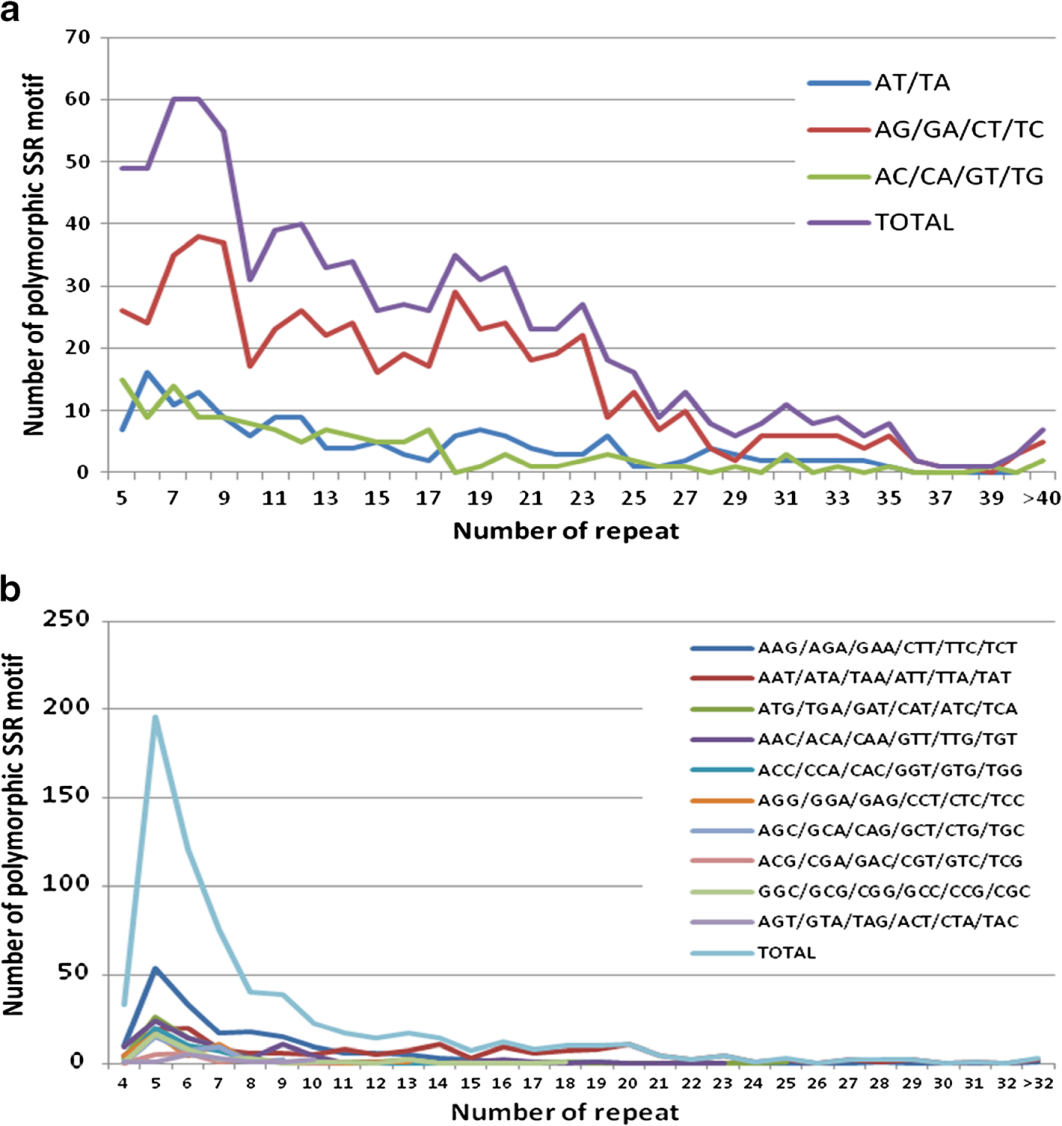
**a Relationship between number of repeat and number of polymorphic SSR motif in dinucleotide SSRs. b** Relationship between number of repeat and number of polymorphic SSR motif in trinucleotide SSRs.

The relationship between the length of an SSR and the frequency of polymorphism was also analyzed by comparing the repeat number of SSRs and the number of polymorphic SSRs (Figure 1). In general, as the repeat number increased, the number of polymorphic SSRs decreased for both dinucleotide and trinucleotide SSRs. The correlation coefficient of the number of polymorphic SSRs with the number of repeat was −0.945 and −0.661 in dinucleotide and trinucleotide SSRs, respectively. In dinucleotide SSRs, repeat number from 5 to 23 (the length between 10 to 46 bp) displayed higher frequencies of polymorphism, i.e. more than 25 polymorphic SSRs for each repeat number within above range. At higher repeat numbers, the frequency dropped to less than 20 polymorphic SSRs. However, in trinucleotide SSRs, repeat number between 4 to 9 (12–27 bp) exhibited more than 40 polymorphic SSRs. At repeat number of more than 10 (30 bp), the number of polymorphic SSRs steeply dropped to less than 25. The peak of frequency of polymorphism for the respective repeat numbers did not always follow the same pattern. For instance, the higher frequency of polymorphism only occurred for repeat number between 4 to 7 (12–21 bp) for motif AAG, while the highest frequency of polymorphism for motif AAT occurred for repeat number 5–6 (15–18 bp), and 14–20 (42–60 bp) in trinucleotide SSRs. Nevertheless, the distribution of polymorphic SSRs among the different repeat numbers was generally skewed to the smaller number of repeats. This might be simply because SSRs with fewer repeats have been identified in high frequencies than those with larger repeat numbers. In common bean, similar result was reported that the number of SSRs was reduced as the repeat number increased in Blair et al. [47]. 

Temnykh et al. [48] provide a threshold number for short and long SSRs, where the length of SSR greater than 20 bp is considered as long SSR, named “class I”; while those less than 20 bp are considered short SSR, named “class II”. Using this criterion, we found 534 SSRs as longer than 20 bp (class I) while 302 SSRs in the short length range (class II) in dinucleotide SSRs. From this point, longer SSRs are more polymorphic than short SSRs although the length of SSR is highly negative correlated with the frequency of polymorphism (correlation coefficient of −0.945). However, in trinucleotide SSRs, the number of long SSRs (333) was similar to short SSRs (339). When considering both dinucleotide and trinucleotide SSRs together, the longer SSRs (867) is indeed greater than short SSRs (635), which is consistent with many previous reports [10,18,43,44].

Increasing availability, affordability and accessibility of molecular markers are facilitating the development of genetic linkage maps in all major crops. Although the first peanut genetic linkage map was reported by [8] using RFLP markers in a wild species x wild species population, no genetic linkage map was developed for cultivated x cultivated peanut until 15 years later when considerable numbers of SSR markers were available. While SSRs have become increasingly important tools for molecular genetic analysis, another potentially useful and widely used marker, Single Nucleotide Polymorphism (SNP), has not been developed yet in peanut.

To date, seven genetic linkage maps have been published for cultivated x cultivated populations using SSR markers [12-14,16-18,49]. Among the 1,343 polymorphic SSRs that we assembled, 593 were mapped in these seven maps (Table 1; Additional file 1). When these maps were constructed, the total available polymorphic SSR markers numbered about six hundred. Therefore, the range of mapped SSR loci in these genetic maps was only from 131 to 324, and these maps still need to be saturated by adding more markers for further molecular research, such as QTL mapping, map-based cloning, and marker-assisted selection in peanut breeding. With a total of 1,343 polymorphic markers available now, including recently generated BAC-end sequence SSRs [18], EST-SSRs [7], and genomic SSRs [36], we presume that construction of a higher density genetic linkage map with ~500 SSR loci in the cultivated peanut is feasible.

Molecular markers are frequently polymorphic in one population, but monomorphic in another. Among the seven genetic linkage maps in cultivated peanut, two maps were constructed using mapping populations from China, three from India, and two from the USA. Some of these informative SSR markers detected polymorphism only in one of three regional populations, but not others, indicating that there is genetic variation between regional populations presumably due to differences in their lineages. However, there were still 45 SSR markers which consistently detected polymorphism across all regional populations of peanuts from China, India and USA. These SSR markers thus may represent the most variable markers so far detected within the peanut genome and corresponding to frequent mutant loci in this crop.

### Conclusions

From an analysis of published literature revealing a total of 9,274 SSR DNA markers in peanut, we identified 1,343 markers detecting polymorphism. The information from such a comprehensive database of polymorphic SSR markers not only facilitates better understanding the nature of SSRs in the peanut genome, but also provides a useful source for conducting additional genetic and genomic studies to improve this crop.

### Availability of supporting data

The data sets supporting the results of this article are included within the article (and its additional file).

## Competing interests

The authors declare that they have no competing interests.

## Authors’ contributions

YZ performed the data analysis, participated in the design of the study and performed the statistic analysis. CSP helped to draft the manuscript and participated in interpretation of data. GH conceived of the study, participated in the design and coordination and drafted the manuscript. All authors read and approved the final manuscript.

## Supplementary Material

Additional file 1 A list of polymorphic SSR markers in peanut. Click here for file

## References

[B1] Kochert G Stalker HT Gimenes M Galgaro SL Lopes CR Moore K RFLP and cytological evidence on the origin and evolution of allotetraploid domesticated peanut, Arachis hypogaea (Leguminosae) Am J Bot 1996 83 1282 1291 10.2307/2446112

[B2] Yuksel B Paterson AH Construction and characterization of a peanut HindIII BAC library Theor Appl Genet 2005 111 4 630 639 10.1007/s00122-005-1992-x 16049705

[B3] Guimarães PM Garsmeur O Proite K Leal-Bertioli SCM Seijo G Chaine C Bertioli DJ D’Hont A BAC libraries construction from the ancestral diploid genomes of the allotetraploid cultivated peanut BMC Plant Biol 2008 8 14 10.1186/1471-2229-8-14 18230166 PMC2254395

[B4] Luo M Dang P Guo BZ He GH Holbrook C Bausher MG Lee RD Generation of Expressed Sequenced tags (ESTs) for gene discovery and marker development in cultivated peanut Crop Sci 2005 45 346 353 10.2135/cropsci2005.0346

[B5] Proite K Leal-Bertioli SC Bertioli DJ Moretzsohn MC da Silva FR Martins NF Guimaraes PM ESTs from a wild Arachis species for gene discovery and marker development BMC Plant Biol 2007 7 7 10.1186/1471-2229-7-7 17302987 PMC1808460

[B6] Guo BZ Chen XP Hong YB Liang XQ Dang P Brenneman T Holbrook C Culbreath A Analysis of gene expression profiles in leaf tissues of cultivated peanuts and development of EST-SSR markers and gene discovery Intl J Plant Genomics 2009 10.1155 10.1155/2009/715605 PMC2703745 19584933

[B7] Koilkonda P Sato S Tabata S Shirasawa K Hirakawa H Sakai H Sasamoto S Watanabe A Wada T Kishida Y Tsuruoka H Fujishiro T Yamada M Kohara M Suzuki S Hasegawa M Kiyoshima H Isobe S Large-scale development of expressed sequence tag-derived simple sequence repeat markers and diversity analysis in Arachis spp Mol Breeding 2011 10.1007/s11032-011-9604-8 PMC3362703 22707912

[B8] Halward TM Stalker HT Kochert G Development of an RFLP linkage map in diploid peanut species Theor Appl Genet 1993 87 379 384 10.1007/BF01184927 24190266

[B9] Burow MD Simpson CE Starr JL Paterson AH Transmission genetics of chromatin from a synthetic amphidiploid to cultivated peanut (Arachis hypogaea L.) broadening the gene pool of a monophyletic Improving Groundnut Farmers' Incomes and Nutrition through Innovation and Technology Enhancement polyploidy species Genetics 2001 159 823 837 11606556 10.1093/genetics/159.2.823 PMC1461827

[B10] Moretzsohn MC Leoi L Proite K Guimaraes PM Leal-Bertioli SCM Gimenes MA Martins WS Valls JFM Grattapaglia D Bertioli DJ A microsatellite-based, gene-rich linkage map for the AA genome of Arachis (Fabaceae) Theor Appl Genet 2005 111 6 1060 1071 10.1007/s00122-005-0028-x 16088397

[B11] Moretzsohn MC Barbosa AVG Alves-freitas DMT Teizeira C Leal-Bertioli SCM Guimaraes PM Pereira RW Lopes CR Cavallari MM Valls JFM Bertioli DJ Gimenes MA A linkage map for the B-genome of Arachis (Fabaceae) and its synteny to the A-genome BMC Plant Biol 2009 9 40 10.1186/1471-2229-9-40 19351409 PMC2674605

[B12] Hong YB Liang XQ Chen XP Liu HY Zhou GY Li SX Wen SJ Construction of genetic linkage map based on SSR markers in peanut (Arachis hypogaea L.) Agricultural Sci in China 2008 7 8 915 921 10.1016/S1671-2927(08)60130-3

[B13] Hong YB Chen XP Liang XQ Liu HY Zhou GY Li SX Wen SJ Holbrook CC Guo BZ A SSR-based composite genetic linkage map for the cultivated peanut (Arachis hypogaea L.) genome BMC Plant Bology 2010 10 17 10.1186/1471-2229-10-17 PMC2835713 20105299

[B14] Varshney RK Bertioli DJ Moretzsohn MC Vadez V Krishramurthy L Aruma R Nigam SN Moss BJ Seetha K Ravi K He GH Knapp SJ Hoisington DA The first SSR-based genetic linkage map for cultivated groundnut (Arachis hypogaea L.) Theor Appl Genet 2009 118 (4 729 739 19048225 10.1007/s00122-008-0933-x

[B15] Fonceka D Hodo-Abalo T Rivallan R Faye I Ndoye M Ndoye O Favero AP Bertioli DJ Glaszmann JC Courtois B Rami JF Genetic mapping of wild introgressions into cultivated peanut: a way toward enlarging the genetic basis of a recent allotetraploid BMC Plant Biol 2009 9 103 10.1186/1471-2229-9-103 19650911 PMC3091533

[B16] Ravi K Vadez V Isobe S Mir RR Guo Y Nigam SN Gowda MVC Radhakrishnan T Bertioli DJ Knapp SJ Varshney RK Identification of several small main-effect QTLs and a large number of epistatic QTLs for drought tolerance related traits in groundnut (Arachis hypogaea L.) Theor Appl Genet 2011 122 1119 1132 10.1007/s00122-010-1517-0 21191568 PMC3057011

[B17] Qin HD Feng SP Chen C Guo YF Knapp S Culbreath A He GH Wang ML Zhang XY Holbrook CC Ozias-Akins P Liang XQ Guo BZ An integrated genetic linkage map of cultivated peanut (Arachis hypogaea L.) constructed from two RIL populations Theor Appl Genet 2011 10.1007/s00122-011-1737-y 22072100

[B18] Wang H Penmetsa RV Yuan M Gong LM Zhao YL Guo BZ Farmer AD Rosen BD Gao JL Isobe S Bertioli DJ Varshney RK Cook DR He GH Development and characterization of BAC-end sequence derived SSRs, and their incorporation into a new higher density genetic map for cultivated peanut (Arachis hypogaea L.) BMC Plant Biol 2012 12 10 10.1186/1471-2229-12-10 22260238 PMC3298471

[B19] Kochert G Halward T Branch WD Simpson CE RFLP variability in peanut (Arachis hypogaea L.) cultivars and wild species Theor Appl Genet 1991 81 565 570 10.1007/BF00226719 24221368

[B20] Halward TM Stalker HT LaRue E Kochert G Use of single-primer DNA amplification in genetic studies of peanut (Arachis hypogaea L.) Plant Mol Biol 1992 18 315 325 10.1007/BF00034958 1731991

[B21] Paik-Ro OG Smith RL Knauft DA Restriction fragment length polymorphism evaluation of six peanut species within the Arachis section Theor Appl Genet 1992 84 201 208 10.1007/BF00224001 24203048

[B22] Subramanian V Gurtu S Rao RCN Nigam SN Identification of DNA polymorphism in cultivated groundnut using random amplified polymorphic DNA (RAPD) assay Genome 2000 43 4 656 660 10.1139/g00-034 10984178

[B23] Hopkins MS Casa AM Wang T Mitchell SE Dean RE Kochert GD Kresovich S Discovery and characterization of polymorphic simple sequence repeats (SSRs) in peanut Crop Sci 1999 39 1243 1247 10.2135/cropsci1999.0011183X003900040047x

[B24] He GH Prakash CS Evaluation of genetic relationship among botanical varieties of cultivated peanut (Arachis hypogaea L.) using AFLP markers Genet Resour Crop Evol 2001 48 347 352 10.1023/A:1012019600318

[B25] Palmieri DA Hoshino AA Bravo JP Lopes CR Gimenes MA Isolation and characterization of microsatellite loci from the forage species Arachis pintoi (Genus Arachis) Molecular Ecology Notes 2002 2 551 553 10.1046/j.1471-8286.2002.00317.x

[B26] He GH Meng RH Newman M Gao GQ Pittman RN Prakash CS Microsatellites as DNA markers in cultivated peanut (Arachis hypogaea L.) BMC Plant Biol 2003 3 3 10.1186/1471-2229-3-3 12713672 PMC155651

[B27] He GH Meng RH Gao H Guo B Gao G Newman M Pittman RN Prakash CS Simple sequence repeat markers for botanical varieties of cultivated peanut (Arachis hypogaea L.) Euphytica 2005 142 131 136 10.1007/s10681-005-1043-3

[B28] Ferguson ME Burow MD Schulze SR Bramel PJ Paterson AH Kresovich S Mitchell S Microsatellite identification and characterization in peanut (A. hypogaea L.) Theor Appl Genet 2004 108 1064 1070 10.1007/s00122-003-1535-2 15067392

[B29] Moretzsohn MC Hopkins MS Mitchell SE Kresovich S Valls JFM Ferreira ME Genetic diversity of peanut (Arachis hypogaea L.) and its wild relatives based on the analysis of hypervariable regions of the genome BMC Plant Biol 2004 4 11 10.1186/1471-2229-4-11 15253775 PMC491793

[B30] Budiman MA Jones JIT Citek RW Warek U Bedell JA Knapp SJ Methylation-filtered and shotgun genomic sequences for diploid and tetraploid peanut taxa http://www.ncbi.nlm.nih.gov/nucgss

[B31] Wang CT Yang XD Chen DX Yu SL Liu GZ Tang YY Xu JZ Isolation of simple sequence repeats from groundnut Electron J Biotechnology 2007 10 3

[B32] Cuc LM Mace ES Crouch JH Quang VD Long TD Varshney RK Isolation and characterization of novel microsatellite markers and their application for diversity assessment in cultivated groundnut (Arachis hypogaea L.) BMC Plant Biology 2008 8 55 10.1186/1471-2229-8-55 18482440 PMC2416452

[B33] Gautami B Ravi K Lakshmi NM Hoisington DA Varshney RK Novel set of groundnut SSRs for genetic diversity and interspecific transferability Int J Integr Biology 2009 7 100 106

[B34] Nagy ED Chu Y Guo YF Khanal S Tang S Li Y Dong WB Timper P Taylor C Ozias-Akins P Holbrook CC Beilinson V Nielsen NC Stalker HT Knapp SJ Recombination is suppressed in an alien introgression in peanut harboring Rma, a dominant root-knot nematode resistance gene Mol Breeding 2010 26 357 370 10.1007/s11032-010-9430-4

[B35] Yuan M Gong LM Meng RH Li SL Dang P Guo BZ He GH Development of trinucleotide (GGC)n SSR markers in peanut (Arachis hypogaea L.) Electron J Biotechnol 2010 13 6

[B36] Macedo SE Moretzsohn MC Leal-Bertioli SCM Alves DMT Gouvea EG Azevedo VCR Bertioli DJ Development and characterization of highly polymorphic long TC repeat microsatellite markers for genetic analysis of peanut BMC Research Notes 2012 5 86 10.1186/1756-0500-5-86 22305491 PMC3296580

[B37] Gimenes MA Hoshino AA Barbosa AVG Palmieri DA Lopes CR Characterization and transferability of microsatellite markers of the cultivated peanut (Arachis hypogaea) BMC Plant Biology 2007 7 9 10.1186/1471-2229-7-9 17326826 PMC1829157

[B38] Naito Y Suzuki S Iwata Y Kuboyama T Genetic diversity and relationship analysis of peanut germplasm using SSR markers Breeding Sci 2008 58 293 300 10.1270/jsbbs.58.293

[B39] Song GQ Li MJ Xiao H Wang XJ Tang RH Xia H Zhao CZ Bi YP EST sequencing and SSR marker development from cultivated peanut (Arachis hypogaea L.) Electronic J Biotechnology 2010 13 3

[B40] Ramsay L Macaulay M Ivanissivich S MacLean K Cardle L Fuller J Edwards K Tuvesson S Morgante M Massari A Maestri E Marniorlin N Sjakste T Ganal M Powell W Powell W Waugh R A simple sequence repeat-based linkage map of barley Genetics 2000 156 1997 2005 11102390 10.1093/genetics/156.4.1997 PMC1461369

[B41] Cordeiro GM Casu R McIntyre CL Manners JM Henry RJ Microsatellite markers from sugarcane (Saccharum spp.) ESTs cross transferable to erianthus and sorghum Plant Sci 2001 160 1115 1123 10.1016/S0168-9452(01)00365-X 11337068

[B42] Song QJ Marek LF Shoemaker RC Lark KG Concibido VC Delannay X Specht JE Cregan PB A new integrated genetic linkage map of the soybean Theor Appl Genet 2004 109 122 128 10.1007/s00122-004-1602-3 14991109

[B43] Burstin J Deniot G Potier J Weinachter C Aubert G Baranger A Microsatellite polymorphism in Pisum sativum Plant Breed 2001 120 311 317 10.1046/j.1439-0523.2001.00608.x

[B44] Mun JH Kim DJ Choi HK Gish J Debelle F Mudge J Denny R Endre G Saurat O Dudez AM Kiss GB Roe B Young ND Cook DR Distribution of microsatellites in the genome of Medicago truncatula: a resource of genetic markers that integrate genetic and physical maps Genetics 2006 172 2541 2555 16489220 10.1534/genetics.105.054791 PMC1456377

[B45] Li YC Korol AB Fahima T Nevo E Microsatellites within genes: structure, function, and evolution Mol Biol Evol 2004 21 6 991 1007 10.1093/molbev/msh073 14963101

[B46] Choudhary OP Trived S Microsatellite or simple sequence repeat (SSR) instability depends on repeat characteristics during replication and repair J Cell and Mol Biol 2010 8 2 21 34

[B47] Blair MW Hurtado N Chavarro CM Munoz-Torres MC Giraldo MC Pedraza F Tomkins J Wing R Gene-based SSR markers for common bean (Phaseolus vulgaris L.) derived from root and leaf tissure ESTs: an integration of the BMc series BMC Plant Biol 2011 11 50 10.1186/1471-2229-11-50 21426554 PMC3068092

[B48] Temnykh S DeClerck G Lukashova A Lipovich L Cartinhour S McCouch S Computational and experimental analysis of microsatellites in rice (Oryza sativa L.): frequency, length variation, transposon association, and genetic marker potential Genome Res 2001 11 1441 1452 10.1101/gr.184001 11483586 PMC311097

[B49] Khedikar VP Gowda MVC Sarvamangala C Patgar KV Upadhyaya HD Varshney RK A QTL study on late leaf spot and rust revealed one major QTL for molecular breeding for rust resistance in groundnut (Arachis hypogaea L.) Theor Appl Genet 2010 121 971 984 10.1007/s00122-010-1366-x 20526757 PMC2921499

